# Mobile device screen time is associated with poorer language development among toddlers: results from a large-scale survey

**DOI:** 10.1186/s12889-024-18447-4

**Published:** 2024-04-15

**Authors:** Signe Boe Rayce, Gunhild Tidemann Okholm, Trine Flensborg-Madsen

**Affiliations:** 1 VIVE– The Danish Center for Social Science Research, Herluf Trolles Gade 11, Copenhagen, 1052 Denmark; 2 Center for Clinical Research and Prevention Bispebjerg and Frederiksberg Hospitals The Capital Region, Nordre Fasanvej 57, Frederiksberg, 2000 Denmark; 3 Unit of Medical Psychology, Department of Public Health, University of Copenhagen, Øster Farimagsgade 5, Copenhagen, 1353 Denmark; 4 The Danish National Institute of Public Health, University of Southern Denmark, Studiestraede 6, Copenhagen, 1455 Denmark

**Keywords:** Child development, Language development, Mobile device screen time, Toddlers

## Abstract

**Background:**

Despite young children’s widespread use of mobile devices, little research exists on this use and its association with children’s language development. The aim of this study was to examine the associations between mobile device screen time and language comprehension and expressive language skills. An additional aim was to examine whether three factors related to the domestic learning environment modify the associations.

**Methods:**

The study uses data from the Danish large-scale survey TRACES among two- and three-year-old children (*n* = 31,125). Mobile device screen time was measured as time spent on mobile devices on a normal day. Measurement of language comprehension and expressive language skills was based on subscales from the Five to Fifteen Toddlers questionnaire. Multivariable linear regression was used to examine the association between child mobile device screen time and language development and logistic regression to examine the risk of experiencing significant language difficulties. Joint exposure analyses were used to examine the association between child mobile device screen time and language development difficulties in combination with three other factors related to the domestic learning environment: parental education, reading to the child and child TV/PC screen time.

**Results:**

High mobile device screen time of one hour or more per day was significantly associated with poorer language development scores and higher odds for both language comprehension difficulties (1–2 h: AOR = 1.30; ≥ 2 h: AOR = 1.42) and expressive language skills difficulties (1–2 h: AOR = 1.19; ≥ 2 h: AOR = 1.46). The results suggest that reading frequently to the child partly buffers the negative effect of high mobile device screen time on language comprehension difficulties but not on expressive language skills difficulties. No modifying effect of parental education and time spent by the child on TV/PC was found.

**Conclusions:**

Mobile device screen time of one hour or more per day is associated with poorer language development among toddlers. Reading frequently to the child may have a buffering effect on language comprehension difficulties but not on expressive language skills difficulties.

**Supplementary Information:**

The online version contains supplementary material available at 10.1186/s12889-024-18447-4.

Children’s language skills help them to develop socially and academically, which implies that the acquisition of language is one of the most important processes in child development. Conversely, language delays and disorders in childhood have been associated with problem behaviors [1], and have also been associated with poorer academic achievement when these children start at school and later in their lives [2–4]. From birth, language develops as the child communicates with their parents and other significant persons [5]. This language development can be enhanced through a cognitively stimulating learning environment such as reading to the child [6, 7].

During the last decade, mobile devices such as tablets and smartphones have become an integrated part of daily life in families. Already from infancy, children are exposed to mobile devices [8–10]. Mobile devices provide children and parents with easy access to entertainment, not only in the home setting but also outside the home. Although this easy access to screen-based entertainment is convenient for parents in some situations [11], it may also pose a risk to toddlers’ language development [12, 13]. Despite the growing exposure of toddlers to mobile devices, little research has focused on mobile device screen time and language development among toddlers [13].

To date, most research on the association between a child’s screen time and language development has focused on either TV or a combination of TV and other digital devices such as smartphones, tablets and computers [13]. These studies have found that children interact less with their parents while the children are watching TV [14, 15], that very young children require adult interaction to learn from screen-based media [7, 16], and that parents are less sensitive to child cues when the parents are engaged in screen use themselves [17]. A study among pre-school children found a negative association between the children’s screen-based media use and white matter microstructural integrity in core areas of the brain supporting language [18]. Although cross-sectional in its design, the findings of this study are in accordance with the results of studies on the association between screen use and language development [13, 19]. In a systematic review and meta-analysis of 38 studies focusing on screen time on various types of devices and language skills among 0-12-year-olds, Madigan et al. (2020) found a negative association between screen time and child language skills. Additionally, they found that the potential benefits of screen-based media were less likely among younger children, although co-viewing and educational content was associated with a small positive effect on child language skills. However, out of the 38 studies included in the review, only two studies focused specifically on mobile device screen time among toddlers [9, 20]. Contrary to Madigan et al. [13], Jing et al. [21] found an overall positive effect of screen media exposure on vocabulary among 0-6-year-old children in a recent meta-analysis of 63 studies. However, in moderator analyses, only experimental studies (testing the effect of exposure to media with an educational content) showed a positive significant effect on vocabulary, with stronger effect found for e-books than apps or games. The meta-analysis comprising studies conducted in a naturalistic setting found no overall effect on vocabulary, with the exception of exposure to media with an educational content. In line with Madigan et al. [13], the meta-analysis by Jing et al. [21] also found larger effects in samples with an average age of 36 months or older.

The effect of screen-based media on language development most likely depends on the content and the context the media are used in [13, 21, 22]. Thus, there are reasons to believe that mobile device screen time may affect language development differently than other screen-based media such as TV and computers. Firstly, mobile devices differ from TV by being portable and therefore easily accessible in various contexts. Secondly, they are handheld, thus co-viewing may be less pronounced [23, 24]. Finally, mobile devices differ from TV by being interactive and by providing reward stimuli [7]. These characteristics may affect child language development either directly, through the content and format which may enhance or inhibit language development, or indirectly, by decreasing the opportunities for children to engage in communicating situations in everyday life [7, 23]. To our knowledge, only few studies have examined the association between mobile device screen time and language development. Of these, two studies suggest a negative association between time spent on mobile devices and expressive language among toddlers [9, 20], while no significant association was found with other communication delays [9] or language comprehension [20]. Another recent study found no significant association between prolonged use of touch screens and overall communication and language development delay among toddlers [25]. The association between mobile device screen time and language development among toddlers is thus unclear.

With child development being multifactorial, the association between mobile device screen time and the child’s language development may also be affected by other activities a child engages in during the day. Research has found that both parental and child screen time is associated with less optimal parent-child communication [7, 14, 15, 17]. However, the parent-child interaction, including language-stimulating activities and book reading, has been found to enhance language development [7, 26, 27]). Thus, Sundqvist et al. (2021) found interactional turn-taking to be a stronger predictor of vocabulary among 25 month old children than TV content and parental device use during daily child routines. Also, the association between mobile device screen time and language development may vary depending on whether the mobile device is the primary unit for screen-based activities or whether the child spends additional time on watching TV thus increasing the overall screen time and risk of missing out on parent-child communication and language stimulating activities [7, 14, 15]. Finally, the overall learning environment in terms of language stimulating activities, norms and attitudes towards screen use in the family may also play a role. These are factors that are associated with parental resources, including parental education [4, 28–30] and may be part of protective or harmful mechanisms [31]. To date, there is, however, a lack of studies that examine the association between mobile device screen time and language development in combination with other factors related to the home and learning environment of the family.

Using a large cross-sectional survey among 31,125 two- and three-year-old children, the objectives of the present study were (1) to examine the association between mobile device screen time and language development among toddlers, and further, (2) to examine this association in combination with three other factors related to the domestic learning environment: parental education, frequency of reading to the child, and child TV/PC screen time. Based on previous research, our hypothesis was that higher mobile device screen time would be associated with poorer language outcomes among toddlers. Additionally, that factors related to domestic learning environment would modify the associations, with a favorable environment reducing the potential negative associations between high mobile device screen time and language development.

Compared to previous studies which have primarily been based on small samples [20, 25], the current study is the largest study to date focusing on mobile device screen time and language development among toddlers. To our knowledge it is also the first to explore this association in combination with other factors related to the domestic learning environment.

## Method

### Study design and population

This study uses cross-sectional baseline data from the Danish longitudinal web-based survey ‘TRACES – Children’s development and well-being throughout life’ [32]. The overall purpose of TRACES is to increase knowledge about small children’s development and well-being and to examine how circumstances and interventions early in life affect well-being and welfare. The baseline survey of TRACES was conducted for VIVE between October, 2017 and September, 2018 by Statistics Denmark and comprises data from 52,010 children aged nine to ten months, two years and three years, equally distributed across the three age groups. Statistics Denmark sampled 90,387 children and subsequently invited the parents of the children to participate in the questionnaire survey through a secure electronic mailbox (e-Boks) the month after their child turned nine months, two years or three years. The questionnaire [33] took approximately half an hour to complete. To accommodate a lower response rate among children from disadvantaged families, children from potentially disadvantaged families were oversampled in the ratio 60/40 in each age group. Potentially disadvantaged families were defined as families where mothers met one or more of the following criteria at child birth: young age, no education other than compulsory school, not cohabiting with the other parent or having received welfare benefits one, two or three years before the birth of the child. Children from the potentially disadvantaged families group were sampled first, and subsequently, a random sample of the remaining children in each age group was sampled. The overall response rate was 57.5% of the sampled children (children from potentially disadvantaged families: 48.4%; other children: 71.1%).

The present study only uses data from the two- and three-year-old children. The study sample was furthermore restricted to the 31,125 children with available data on both language development and mobile device screen time (90.6%). This sample is close to representative of the full population of children in Denmark in these two age groups, in that the sample displays only minor differences in child age, parental education, employment status and parental migration status when compared with the full population of children in Denmark in these two age groups (see supplementary table S1).

### Measures

#### Child mobile device screen time

Child mobile device screen time was measured by the item “On a normal weekday, how much time does [name of the child] spend on an iPad, tablet or smartphone (e.g. playing games or watching a movie)?” The response categories were: “No time at all”, “less than half an hour a day”, “half to 1 hour a day, “1–2 hours a day” and “More than 2 hours a day”. In order to provide hourly intervals, “less than half an hour” and “half to 1 hour” were collapsed, and sensitivity analyses showed no substantial differences in results when this was done.

#### Language development

Measurement of language comprehension and expressive language skills was based on two subscales from the parent-reported Five to Fifteen Toddlers questionnaire (FTF-Toddlers) [34]. This questionnaire is a toddler version (two to five years) of the Five to Fifteen questionnaire (FTF), which is a Nordic questionnaire for evaluation of development and behavior, including language development, among five- to fifteen-year-olds [35]. While the FTF has been validated in various settings [36], the FTF-Toddlers is still being tested [37]. The items of the two subscales are statements about the child’s language development (see supplementary tables S2 and S3 for items). The parent is asked to report how they feel that their child functions compared to children of the same age on a three-point Likert-type scale: “Does not apply” (0), “Applies sometimes/to some extent” [1] and “Applies” [2]. Sum scores are calculated for each subscale, with higher scores indicating poorer language development.

Language comprehension was measured by the seven-item FTF-Toddlers language comprehension scale (e.g. “Has difficulty understanding words” and “Has difficulty understanding simple instructions”) with a score range of 0–14 and a Cronbach’s α of 0.83. While the FTF language comprehension scale was unidimensional, initial exploratory factor analysis (iterated principal factor analysis with oblique (promax) rotation) pointed towards a two-dimensional structure of the ten-item FTF-Toddlers spoken language subscale reflecting: (1) expressive language skills (items 61–65) and (2) more speech-related aspects such as stuttering and voice usage (items 66–70) (see supplementary tables S3 and S4). Based on conceptual considerations and unidimensionality being a fundamental requirement of psychometric scales we omitted items on speech related aspects in the present study. Instead we focused on expressive language skills measured by a five-item scale comprising items such as “Has difficulties saying single words and short sentences” and “Has difficulty speaking so that his/her parents understand him/her” (score range: 0–10, Cronbach’s α: 0.86).

Besides the two scales on language development, two dichotomous variables of difficulties with language comprehension and expressive language skills were used in the logistic regression analyses. Currently, no norms for the FTF-Toddlers exist. However, the sample used in the present study is close to representative for the Danish population of 2- and 3-year olds (supplementary table S1). A recent systematic review have found a prevalence of low language skills to vary between 3.4% and 17.5% (M = 10.7%) in children aged between 1 and 4 years [38]. Therefore, scores at or above the 90th percentile in the child’s gender and age group were used as cut-offs for being *at risk* of language difficulties (hereafter: difficulties) (see supplementary table S5). This is in agreement with the procedure used in studies using the original FTF questionnaire where a score above the 90th percentile marks the threshold for clinical concern [39].

#### Covariates

Child gender, child age, parental education, parental employment status, and parental migration status were based on administrative data from Statistics Denmark. *Parental education* was measured as the highest achieved education of the parents and categorized into short (short-cycle higher education, high school or less), medium (medium-cycle higher education) and long education (long-cycle higher education). *Parental employment status* was measured as the number of parents in employment. Because of known differences in child-rearing practices among different cultures, also when it comes to screen time use, *parental migration status* was included in the analyses [6]. Parental migration status was classified according to the standards of Statistics Denmark [40] and categorized into: (1) both parents of Danish origin, (2) one parent of Danish origin and one either immigrant or descendant of immigrants, and (3) both parents being immigrants or descendants of immigrants.

Parental mental well-being, parental preoccupation with smartphone, reading stories to the child and child screen time on TV or PC were assessed in the survey. *Parental mental well-being* was measured by WHO-5 [41], with a score below 50 indicating risk of depression or stress. *Parental preoccupation with smartphone* as a proxy for smartphone use was measured by a 0–2 point index based on two dichotomized indicators of being highly preoccupied with one’s smartphone: “Thinking of the mobile phone when not using it” and “frequently checking the mobile phone so as not to miss out on what is going on” (indication of high preoccupation for both statements was signaled by the answers “highly true” or “completely true” (coded 1), while no indication of high preoccupation was signaled by the answers “Not at all true”, “a little bit true”, “somewhat true” or “true to a large extent” (coded 0)) ). *Child screen time* on TV/PC was measured by the item “On a normal weekday, how much time does [name of the child] spend watching TV or movies on the TV or on a computer?” with response categories coded similarly to mobile device screen time. Finally, *Reading stories* to the child in the past month [42] was dichotomized in order to create an indicator for frequent (on “a daily” or “almost daily” basis) and infrequent reading (“a few times a week”, “a few times a month” “rarely” or “not at all”), respectively.

### Statistical analyses

Multivariable linear regression with mobile device screen time treated as a categorical variable was used to examine the association between child mobile device screen time and language comprehension and expressive language skills score. In order to examine how mobile device screen time was associated with indication of risk of language difficulties relative to the majority of children in the same age and gender group we used logistic regression. Logistic regression was used to estimate odds ratios (ORs) for language development scores at or above the 90% percentile in the child’s age and gender group. The analyses were conducted in three steps: a crude model (Model 1), a model adjusted for sociodemographic variables (Model 2), and a full model where factors related to the child’s home environment (parental mental well-being, parental preoccupation with smartphone, screen time on TV/PC and reading to the child) were added (Model 3). This approach was chosen in order to disentangle the effect of adjusting for sociodemographic variables and additionally the home environment. All included confounders were significantly associated with both mobile device screen time (chi^2^-test, *p* < 0.001), and language comprehension and expressive language skills (Mood’s median test, *p* < 0.001).

In accordance with STROBE recommendations, the potential modifying effect of three dichotomized factors related to the learning environment of the home (parental education, frequency of reading to the child and child TV/PC screen time, respectively) was determined using joint exposure analyses [43]. In this way not only the separate effects of high mobile device screen time and the dichotomized home environment factor on language development but also the joint effect of the two variables can be examined. Thus, it is possible to identify protective or harmful combinations of screen time and the included factors related to the learning environment. In the joint exposure analyses, high mobile device screen time was defined as ≥ 1 h based on the guidelines by WHO [44] and the American Academy of Pediatrics [45]. Thus, children with low mobile device screen time and no exposure in TV/PC screen time, frequency of being read for or whose parents’ highest education was categorized as short, respectively were considered the reference group in the three joint exposure analyses. Taking a public health perspective [43], test for effect modification was defined as departure from additivity and was conducted using Rothman’s method for creating interaction terms [46]. Synergy indexes (SI) adjusted for confounding were calculated by the equation (OR_EXP++_ - 1)/([OR_EXP+−_ -1] + [OR_EXP−+_ -1]) with 95% confidence intervals calculated according to the methods described by Hosmer and Lemeshow [47]. A SI can go from 0 to infinity, where a SI of 1 (exactly additivity) means no effect modification i.e. the combined effect of the two exposures being equal to the sum of the absolute effects of the two exposures. A SI above 1 means more than additivity of the effects of the two exposures, i.e. the combined effect being larger than the sum of the absolute effects of the two exposures. A SI below 1 means less than additivity of the two effects. All analyses were conducted using the statistical software SAS version 9.4.

### Missing data

Out of the full study sample of 31,125 children, a total of 28,141 children (90.4%) had complete data on all covariates included in the fully adjusted model 3. Only few significant differences were found between the full study sample and the subsample of children with complete data. Children with complete data were significantly less likely to come from a family with short educations (41% vs. 42% in the full study sample) and families where both parents were immigrants or descendants of immigrants (13.5% vs. 14.1%) (See supplementary table S1). The analyses were based on the full study sample. Complete case analyses (*n* = 28,141) of model 1 and model 2 revealed comparable results.

## Results

Descriptive statistics are presented in Table 1. The sample was evenly divided in children aged 2- and 3 years. Likewise, around half of the sample were girls (48.9%). A total of 42.0% had parents with a short education and 6.4% of the children came from families with two unemployed parents. The majority of children (74.7%) had two parents with Danish origin while 14.1% had two parents who were immigrants or descendants of immigrants. Overall, 72.2% of the children spent time on mobile devices during a normal weekday (i.e. the five weekdays Monday to Friday). Most children (62.2%) spent less than one hour on mobile devices, while 10% spent one hour or more. In comparison, 88% of the children spent time on the TV/PC, with 75.5% using the TV/PC for less than one hour. The distributions of language comprehension and expressive language skills in the sample were positively skewed for both 2- and 3- year old children (see Table 2).

**Table 1:** Sample characteristics: (1) frequency of exposure, and covariates in the sample and (2) prevalence of mobile device screen use ≥ 1 h by covariates

	Study sample (*n* = 31,125)	≥ 1 h mobile device screen time during a normal weekday	*p*-value^a^
	n (%)	(%)	
Age (*n* = 31,125)			*p* < 0.001
2 years	15,472 (49.7)	7,5	
3 years	15,653 (50.3)	12.3	
Child gender (*n* = 31,125)			*p* = 0.002
Male	15,916 (51.1)	10.4	
Female	15,209 (48.9)	9.4	
Family educational level (*n* = 30,635)			*p* < 0.001
Short^b^	12,851 (42.0)	11.8	
Medium^c^	8584 (28.0)	9.3	
Long^d^	9200 (30.3)	7.6	
Parental employment status (*n* = 28,613)			*p* < 0.001
Both parents work	19,000 (66.4)	8.6	
One parent works	7780 (27.2)	12.3	
No parents work	1833 (6.4)	13.3	
Migration status (*n* = 28,741)			*p* < 0.001
Both parents of Danish origin	21,455 (74.7)	8.6	
One parent of Danish origin + one parent immigrant or descendant of immigrants	3240 (11.3)	9.7	
Both parents immigrants or descendants of immigrants	4046 (14.1)	16.9	
Mobile device screen time during a normal weekday (*n* = 31,125)			
No screen	8664 (27.8)	-	
< 1 h	19,373 (62.2)	-	
1–2 h	2669 (8.6)	-	
> 2 h	419 (1.4)	-	
TV/PC screen time during a normal weekday (*n* = 31,114)			*p* < 0.001
No screen	3732 (12.0)	13.4	
< 1 h	22,251 (71.5)	5.7	
1–2 h	4471 (14.4)	23.7	
> 2 h	660 (2.1)	40.6	
Reading to the child (*n* = 31,125)			*p* < 0.001
Daily or almost daily	20,213 (64.9)	7.7	
A few times a week or less frequently	10,912 (35.1)	14.1	
Mental well-being (*n* = 31,125)			*p* < 0.001
High (WHO-5: ≥50)	27,001 (86.8)	9.5	
Low (WHO-5: <50)	4124 (13.3)	12.8	
Parental preoccupation with smartphone (*n* = 30,979)			*p* < 0.001
0 indicators of high preoccupation with smartphone	20,291 (65.5)	8.6	
1 indicator of high preoccupation with smartphone	8421 (27.18)	11.7	
2 indicators of high preoccupation with smartphone	2267 (7.32)	15.0	

^a^chi^2^-test, ^b^short-cycle higher education, vocational school, high school or less, ^c^medium-cycle higher education, ^d^long-cycle higher education. IQR = Interquartile range. Two variables had more than 5% missing data: Family educational level (8.1%), Parental employment status (7.7%)

Table 1 also shows the prevalence of ≥ 1 h mobile device screen time by covariates. Mobile device screen time was significantly associated with all sociodemographic characteristics, with screen time of one hour or more being more prevalent among children from families with short educations, among children from families with a lower attachment to the labor market and among children from families where both parents were immigrants or descendants of immigrants. Likewise, all factors related to the home environment were significantly associated with mobile device screen use. Thus, mobile device screen time of one hour or more was more prevalent among children with higher screen time on TV/PC, among children being read to a few times a week or less frequently, among children having a parent with low mental well-being and among children of parents with indicators of high preoccupation with smartphone.

The distribution of language comprehension skills and expressive language skills differed significantly between children with mobile device screen time of one hour or more and children with less screen time (Mood’s median test, *p* < 0.001) with higher median scores among children with mobile device screen time of one hour or more (Table 2).

**Table 2:** Distribution of the language domains presented as (1) median scores and interquartile ranges (IQR) among 2- and 3-year old boys and girls and (2) and frequency of children of risk of difficulties

	< 1 h mobile device screen time during a normal weekday				≥ 1 h mobile device screen time during a normal weekday				Difference between medians
	n	Median	IQR	Children at risk of difficulties n (%)^b^	n	Median	IQR	Children at risk of difficulties/ n (%)^b^	p^a^
Language comprehension									
2-year old boys	7310	2	(1–4)	615 (8.4)	608	3	(1–5)	110 (18.1)	< 0.001
2-year old girls	7003	1	(0–3)	818 (11.7)	551	2	(1–4)	119 (21.6)	< 0.001
3-year old boys	6946	1	(0–2)	717 (10.3)	1052	1	(0–3)	219 (20.8)	< 0.001
3-year old girls	6778	0	(0–1)	573 (8.4)	877	1	(0–2)	147 (16.8)	< 0.001
Expressive language skills									
2-year old boys	7310	2	(1–5)	700 (9.6)	608	3	(2–6)	104 (17.1)	< 0.001
2-year old girls	7003	2	(1–4)	777 (11.1)	551	2	(1–4)	93 (16.9)	< 0.001
3-year old boys	6946	1	(0–2)	634 (9.1)	1052	1	(0–3)	172 (16.3)	< 0.001
3-year old girls	6778	0	(0–2)	586 (8.6)	877	1	(0–3)	151 (17.2)	< 0.001

^a^Mood’s Median Test, ^b^Se Supplementary table S5 for 90^th^percentiles cut−off scores

### Mobile device screen time and language development

Table 3  presents the results of the linear regression analyses with positive B-coefficients indicating poorer language development. For both language comprehension and expressive language skills, spending one hour or more on mobile devices on normal weekdays was associated with poorer language development. Compared to children with no mobile device screen time, the crude B-coefficients (Model 1) for children spending more than two hours on mobile devices were 1.36 (95% CI: 1.13, 1.60) for language comprehension and 0.99 (95% CI: 0.76, 1.23) for expressive language skills. After first adjusting for sociodemographic characteristics (Model 2) and then further adjusting for home environment characteristics (Model 3), B-coefficients decreased markedly for children spending more than two hours on mobile devices, but remained significant. Thus, the average language comprehension score of children who spent more than two hours on mobile devices was 0.48 higher (95% CI: 0.23, 0.73) (on a scale ranging from 0 to 14) compared to the score of children with no mobile device screen time, indicating poorer language development among children with higher mobile device screen time. For expressive language skills, the corresponding B-coefficient was 0.46 (95% CI: 0.21, 0.71) on a scale ranging from 0 to 10. Spending less than one hour on mobile devices was associated with a slightly better language comprehension and expressive language skills score, but the estimates diminished with adjustment for sociodemographic characteristics and was no longer significant for expressive language skills.

**Table 3:** Crude and adjusted regression coefficients for language comprehension and expressive language skills development

	Language comprehension B (95% CI)			Expressive language skills B (95% CI)		
	Model 1 Crude (*n* = 31,125)	Model 2 Adjusted^a^ (*n* = 28,273)	Model 3 Fully adjusted^b^ (*n* = 28,141)	Model 1 Crude (*n* = 31,125)	Model 2 Adjusted^a^ (*n* = 28,273)	Model 3 Fully adjusted^b^ (*n* = 28,141)
Screen time on mobile devices during a normal weekday						
No screen (reference)	0	0	0	0	0	0
< 1 h	-0.13*** (-0.20,-0.07)	-0.04 (-0.10, 0.02)	-0.06* (-0.12, 0.00)	-0.24*** (-0.29, -0.17)	-0.05 (-0.11, 0.01)	-0.06 (-0.11, 0.00)
1–2 h	0.49*** (0.39, 0.60)	0.46*** (0.35, 0.56)	0.27*** (0.16, 0.38)	0.24*** (0.14, 0.35)	0.41*** (0.31, 0.52)	0.27*** (0.16, 0.37)
> 2 h	1.36*** (1.13, 1.60)	0.92*** (0.68, 1.16)	0.48*** (0.23, 0.73)	0.99 (0.76, 1.23)	0.85 (0.61, 1.09)	0.46*** (0.21, 0.71)

* *p* < 0.05, **p < 0.01, ***p < 0,001

^a^ Adjusted for sociodemographic characteristics: child gender and age, parental education, employment and migration status

^b^ Adjusted for sociodemographic characteristics and factors related to the home environment of the child: child gender and age, parental education, employment, migration status, mental well-being, preoccupation with smartphone and reading to the child and child screen time on TV/PC

### Mobile device screen time association with language comprehension difficulties and expressive language skills difficulties

Table 4 presents the crude and adjusted odds ratios for having difficulties with language comprehension and expressive language. For both outcomes, a significant association between mobile device screen time of one hour or more and being at risk of having language development difficulties was found. Children spending more than two hours on mobile devices during a normal weekday had an OR of 3.68 (95% CI: 2.94, 4.60) for language comprehension difficulties and an OR of 3.12 (95% CI: 2.48, 3.92) for difficulties with expressive language skills compared to children with no screen time (Model 1). When adjusted for sociodemographic and home environment characteristics (Model 3), these ORs decreased markedly to 1.42 (95% CI: 1.08, 1.88) for language comprehension and to 1.46 (95% CI: 1.10, 1.93) for expressive language skills difficulties. For the category of less than one hour of mobile device screen time, a slightly lower OR for expressive language skills difficulties was found when compared to children with no screen time.

**Table 4:** Crude and adjusted odds ratios for language comprehension and expressive language skills difficulties

	Language comprehension difficulties OR (95% CI)			Expressive language skills difficulties OR (95% CI)		
	Model 1 Crude (*n* = 31,125)	Model 2 Adjusted^b^ (*n* = 28,273)	Model 3 Fully adjusted^c^ (*n* = 28,141)	Model 1 Crude (*n* = 31,125)	Model 2 Adjusted^b^ (*n* = 28,273)	Model 3 Fully adjusted^c^ (*n* = 28,141)
Mobile devices screen time during a normal weekday						
No screen (reference)	1	1	1	1	1	1
< 1 h	0.98 (0.90, 1.07)	0.93 (0.85, 1.02)	0.91 (0.83, 1.01)	0.89** (0.81, 0.96)	0.88*** (0.81, 0.97)	0.88** (0.80, 0.96)
1–2 h	1.98*** (1.76, 2.24)	1.57*** (1.37, 1.80)	1.30*** (1.13, 1.49)	1.57*** (1.38, 1.78)	1.38*** (1.20, 1.58)	1.19* (1.03, 1.37)
> 2 h	3.68*** (2.94, 4.60)	2.16*** (1.68, 2.80)	1.42* (1.08, 1.88)	3.12*** (2.48, 3.92)	2.03*** (1.57, 2.63)	1.46** (1.10, 1.93)

* *p* < 0.05, **p < 0.01, ***p < 0,001

^a^Defined as a score at or above the 90th percentile on the respective language development scales

^b^Adjusted for child gender and age, parental education, employment and migration status

^c^Adjusted for child gender and age, parental education, employment, migration status, mental well-being, preoccupation with smartphone, reading to the child and child screen time on TV/PC

### Joint exposure analyses of mobile device screen time and factors related to the domestic learning environment

Table 5 presents the results of the analyses combining mobile device screen time with parental education, reading to the child, and child TV/PC screen time. Compared to children with low mobile device screen time and whose parents had medium/long educations, children of parents with short education (OR = 1.72; 95% CI: 1.57, 1.88) or exposed to high mobile device screen time (OR = 1.93; 95% CI: 1.64, 2.27) were at higher risk of language comprehension difficulties. The corresponding ORs for expressive language skills difficulties were 1.30 (95% CI: 1.19, 1.41) among children of parents with short parental education and 1.72 (95% CI: 1.47, 2.02) when exposed to high mobile device screen time. For both types of language development difficulties, the ORs increased when exposed to both risk factors. However, the test for effect modification did not support departure from additivity.

Compared to children with low screen time on both TV/PC and mobile devices, children with either high screen time (≥ 1 h) on TV/PC (OR = 1.51; 95% CI: 1.35, 1.68) or high mobile device screen time (OR = 1.91; 95% CI: 1.65, 2.20) were both at higher risk of language comprehension difficulties, with a tendency towards the strongest association between mobile device screen time and difficulties. Likewise, the corresponding ORs for expressive language skills difficulties were 1.38 (95% CI: 1.23, 1.54) when exposed to high screen time on TV/PC and 1.71 (95% CI: 1.48, 1.98) when exposed to high mobile device screen time. For both outcomes, the ORs for difficulties increased when the children were exposed to both high mobile device screen time and high TV/PC screen time, but the test for effect modification did not support departure from additivity.

Compared to children with less than one hour mobile device screen time who were read to on a daily or almost daily basis, children exposed to either infrequent reading (OR = 1.52; 95% CI: 1.27, 1.82) or high mobile device screen time (OR = 2.13; 95% CI: 1.95, 2.32) were at higher risk of language comprehension difficulties. When exposed to both high mobile device screen time and infrequent reading, the OR for language difficulties increased markedly to 3.49 (95% CI: 3.03, 4.02) for language comprehension difficulties. The test for effect modification supported departure from additivity (SI = 1.51; 95% CI: 1.17, 1.93), with the OR of combined exposure of high mobile device screen time and infrequent reading being more than additive. For expressive language skills difficulties, the ORs were 1.55 (95% CI: 1.30, 1.84) when exposed to high mobile device screen time only and 1.74 (95% CI: 1.60, 1.90) when exposed to infrequent reading only. Combined, the OR for expressive language skills difficulties increased, but the test for effect modification did not support departure from additivity.

**Table 5:** Adjusted odds ratios and synergy indexes (95% CI) for language comprehension and expressive language skills difficulties by combination of mobile device screen time and factors related to the domestic learning environment

	Language comprehension difficulties OR (95% CI) (*n* = 28,141)	Expressive language skills difficulties OR (95% CI) (*n* = 28,141)
Mobile device screen time^a^ and parental education^b^		
Low mobile device screen time and medium/long education	1	1
Low mobile device screen time and short education	1.72*** (1.57, 1.88)	1.30*** (1.19, 1.41)
High mobile device screen time and medium/long education	1.93*** (1.64, 2.27)	1.72*** (1.47, 2.02)
High mobile device screen time and short education	2.80*** (2.41, 3.25)	1.94*** (1.65, 2.27)
Synergy index (SI)	SI:1.09 (0.83, 1.44)	SI: 0.92 (0.62, 1.37)
Mobile device screen time^a^ and child TV/PC screen time^a, c^		
Low mobile device screen time and low TV/PC screen time	1	1
Low mobile device screen time and high TV/PC screen time	1.51*** (1.35, 1.68)	1.38*** (1.23, 1.54)
High mobile device screen time and low TV/PC screen time	1.91*** (1.65, 2.20)	1.71*** (1.48, 1.98)
High mobile device screen time and high TV/PC screen time	2.20*** (1.89, 2.56)	1.87*** (1.59, 2.19)
Synergy index (SI)	SI: 0.85 (0.61, 1.19)	SI: 0.79 (0.53, 1.20)
Mobile device screen time^a^ and frequency of reading to the child^d, e^		
Low mobile device screen time and frequent reading	1	1
Low mobile device screen time and infrequent reading	2.13*** (1.95, 2.32)	1.74*** (1.60, 1.90)
High mobile device screen time and frequent reading	1.52*** (1.27, 1.82)	1.55*** (1.30, 1.84)
High mobile device screen time and infrequent reading	3.49*** (3.03, 4.02)	2.52*** (2.17, 2.93)
Synergy index (SI)	SI: 1.51*** (1.17, 1.93)	SI: 1.18 (0.87, 1.61)

* *p* < 0.05, **p < 0.01, ***p < 0,001

^a^High: ≥1 h during a normal weekday vs. low: <1 h during a normal weekday

^b^Adjusted for child gender and age, parental employment, migration status, mental well-being, preoccupation with smartphone, reading to the child and child screen time on TV/PC

^c^Adjusted for child gender and age, parental education, employment, migration status, mental well-being and preoccupation with smartphone, and reading to the child

^d^Frequent: daily or almost daily vs. infrequent: two times a week or less

^e^Adjusted for child gender and age, parental education, employment, migration status, mental well-being, preoccupation with smartphone and child screen time on TV/PC

## Discussion

The current study suggests that high mobile device screen time is associated with poorer language comprehension and expressive language skills among two- and three-year-old children. Children with mobile device screen time of one hour or more had significantly poorer language development scores on both outcomes compared to children with no mobile device screen time. Likewise, higher odds for being at risk of language comprehension and expressive language skills difficulties were found even after adjustment for sociodemographic characteristics and factors related to the home environment. This confirms the significance of the findings in terms of indication of significant difficulties relative to the majority of children in the same age and gender group. However, mobile device use of less than one hour a day was not associated with poorer language development on any of the two language development measures. Neither was it associated with increased odds for risk of language comprehension and expressive language skills difficulties, respectively. Finally, the results indicate that one aspect of the domestic learning environment– reading frequently to the child– may be a moderating factor that partly buffers the negative effect of high mobile device screen time on language comprehension. However, no modifying effect of parental education and time spent by the child on TV/PC was found.

Our results are in accordance with findings of previous studies examining the association between overall screen time and language development [13, 48] and with a recent longitudinal study that found high screen use in early childhood to be associated with poorer language and cognitive development in kindergarden [49]. Contrary to the findings of the recent meta-analysis by Jing et al. [21], our study found poorer language development scores and higher risks of both language comprehension difficulties and expressive language skills difficulties among 2- and 3-year olds with a high mobile device screen time. There may be a number of reasons for the difference in findings. First, the overall meta-analysis by Jing et al. [21] included both experimental studies and studies conducted in a naturalistic setting. When moderator analyses were conducted, only experimental studies (testing the effect of screen media with educational content) and studies with naturalistic exposure to educational content showed a positive association between media exposure and vocabulary. No significant association was found for studies conducted in ‘real world’ settings where a greater diversity in screen content and quality can be expected. Secondly, a stronger effect was found when studies assessed learning of specific words taught as part of the experiment rather than assessing general vocabulary. Finally, both Jing et al. [21] and Madigan et al. [13] found that younger children benefit less from screen media exposure. Our findings may therefore partly be due to the younger age of the children comprised in our sample (mean age: 31 months) compared to the meta-analysis by Jing et al. [21] (mean age experimental setting: 49 months; naturalistic setting: 33 months). In relation to the association between mobile device screen time, specifically and language development, our findings are consistent with the study of van den Heuvel et al. [9] who found negative associations between mobile device screen time and expressive language among 18-month-old children. Likewise with Moon et al. [20], who found negative associations between mobile device screen time and expressive language among three-year-olds. However, contrary to our study, Moon et al. [20] found no association with language comprehension, and van den Heuvel [9] found no association between mobile device screen time and other communication delays (social and symbolic communication including for example gestures and understanding) among 18-month-olds. In addition, another study by Lin et al. [25] found no association between time spent on mobile devices and overall language delay (combining language and communication skills) among 18- to 36-month-olds. Research in the area is thus often inconsistent, which is most likely due to different measures and operationalizations of both screen use and language development. Thus, especially language comprehension has been operationalized in various ways and additionally, the mode of data collection differed regarding whether completion of the questionnaire was supported by a professional [20, 25] or not. Likewise, the operationalization of mobile device screen time also differed with regard to the questions being asked, and how the response categories were presented and coded. Therefore, differences between the results of the present study and previous studies focusing specifically on mobile device screen time may be partly due to issues related to measurement and operationalization. Additionally, cultural issues related to the child’s environment may have impacted results from different countries; in some environments there may for example be moderating factors not accounted for.

### Potential mechanisms

Some potential mechanisms may contribute to that high mobile device screen time is associated with poorer language comprehension and expressive language skills. One mechanism could be time spent on mobile devices may replace time that could be spent on parent-child interaction and language enhancing activities [7, 23, 50]. In addition, the content that young children engage with on mobile devices vary in quality and may in some cases be age-inappropriate [13, 21]. Another mechanism could be that some parents may perceive educational apps, for example, as an alternative to more traditional learning activities which are more beneficial for language development [51, 52]. However, this potential mechanism may be less likely as Levine et al. [53] find that parents with an educational motive for letting their toddler spend time on mobile devices are more likely to co-view the content. If children with high screen time miss out on important communication opportunities with parents or significant others such as siblings, these missed communication opportunities may affect the children’s language development negatively [29, 54].

One way to support parent-child interaction and child language development is through shared book reading, which provides opportunities for dialogue and elaborations of concepts and events [26]. Additionally, shared book reading provides the child with an opportunity to explore the story line and rehearse new words at her or his own pace. In line with this, we found that frequent reading to the child may act as a buffer by reducing some of the negative effect of high mobile device screen time on language comprehension difficulties. However, no modifying effect of reading was found for expressive language skills difficulties. This may be due to the fact that reading for some parents is a one-way communication in which the child listens but only to a limited degree express words itself [31].

Contrary to our expectations, two other factors related to the domestic learning environment– parental education and time the child spent on watching TV or PC– did not modify the association between high mobile device screen time and language development. This suggests two independent effects: mobile device screen time and parental education appear to affect language development independently of one another; and mobile device screen time and TV/PC time appear to affect language development independently of one another. That is, the OR of the combined exposures did not exceed the sum of the separate ORs.

### Strength and limitations

A major strength of the present study is the large, close-to-representative sample of two- and three-year-old children that provides data on mobile device screen time, child language development, parental characteristics and the child’s home environment. This made it possible to adjust for important confounders such as parental mental well-being, parental preoccupation with smartphone and frequency of reading to the child, and to examine potential modifiers of the association. Secondly, measuring mobile device screen time and time spent on TV/PC separately enabled us to study the association between language development and mobile device screen time specifically. To our knowledge, only few studies have studied this association despite the widespread use of mobile devices among toddlers and despite the shift towards use of mobile devices instead of TV and PCs (which most research focus on) during the last decade [13].

There are, however, also a number of important limitations in the present study. First, the cross-sectional design of the study does not reveal the direction of the association between mobile device screen time and language development. Secondly, both mobile device screen time and language development were parent reported. This may have caused screen time to be underreported if some parents wanted to provide a more socially desirable answer. However, parents were informed that their response to the web-based questionnaire was treated as strictly confidential before completing the questionnaire. Thirdly, assessment of language development is complex. Therefore, the FTF-Toddlers questionnaire cannot be used for diagnostic determination of language problems since such diagnostic determination should always be conducted by a professional. Also, detailed information on language development such as vocabulary and grammar were not available, which may have given us a less nuanced picture of children’s language development. Still, parents experience their child in various contexts. Since the FTF-Toddlers subscales are based on these parental experiences, the subscales do provide information on parent-reported difficulties within language development. We did not find any systematic differences in the findings of previous studies on mobile device screen time and language development [9, 20, 25], depending on outcome measure nor whether language development was parent reported or directly assessed by a professional. Another limitation is that the association between mobile device screen time and language development may depend on the content quality, degree of co-viewing and interaction about the content. For example, co-viewing has been stressed as important for young children, because they rely on interaction with their adult caregivers in order to transfer learning achieved from screens to real-world settings [7, 16]. Likewise, the effect of mobile device screen use may depend on whether the content is age appropriate and of an educational character [13, 21]. Also, children whose parents let their children use mobile devices for non-educational purposes may be more likely to spend time alone on mobile devices [53]. While the need for more knowledge of co-viewing and content quality has been highlighted [9, 13], we did not have information about these factors in the present study. Finally, frequency of reading to the child was included as a potential confounder in the analyses of the association between mobile device screen time and language development. However, it is possible that frequency of reading is a mediator between screen time and language development, that is, that parents spend less time reading to their child because the child asks for and spends time on mobile devices. If so, including reading as a confounder would be an over adjustment and the associations of the present study would be underestimated.

### Conclusions and implications

Mobile devices have become an integral part of everyday life and they are now ubiquitous in the lives of even very young children. This study found that one hour or more of mobile device screen time a day was significantly associated with poorer language development scores and higher risks of both language comprehension difficulties and expressive language skills difficulties among two- and three-year-old children. The results remained significant after adjustment for sociodemographic and home environment characteristics. A buffering effect of reading to the child was found on language comprehension difficulties, while parental education and child TV/PC time did not modify the associations with language comprehension difficulties or with expressive language difficulties.

Since the study is cross-sectional, further research using longitudinal data is needed in order to determine the causality of our findings. However, our results point out significant associations between high mobile device use and poorer language development, which in itself is associated with socio-emotional, educational and cognitive outcomes [1–4, 55]. The knowledge of this study is therefore of importance to both parents, child specialists and clinicians as it illuminates the potential risks of providing young children with mobile devices to a degree that it may negatively affect their language development.

### Electronic supplementary material

Below is the link to the electronic supplementary material.

Supplementary Material 1

Supplementary Material 2

Supplementary Material 3

Supplementary Material 4

Supplementary Material 5

## Data Availability

Data used in this study are not publicly available as they are stored on the secure servers of Statistics Denmark. Due to privacy concerns, the data cannot be made available outside the hosted research servers at Statistics Denmark. Provided that researchers are affiliated with a research environments preapproved by Statistics Denmark, data are available on reasonable request sent to the corresponding author.
