# Supplementary material for: Mobile device screen time is associated with poorer language development among toddlers: results from a large-scale survey
Source: BMC Public Health. 2024 Apr 15;24:1050. doi: 10.1186/s12889-024-18447-4 (PMC11020890; doi:10.1186/s12889-024-18447-4)
Supplement: Supplementary file 1 — Supplementary Material 1 [file 12889_2024_18447_MOESM1_ESM.docx]

**Supplementary table S1**

*Sociodemographic characteristics of 1) the full population of Danish two- and three-year-olds, 2) the study sample and 3) study sample with full information on all variables*

|  | Full population of two- and three-year-old children in Denmark  ( n=122901) | Study sample^a^  (n=31125) | Difference between study sample and national sample  p-value^b^ | Study sample with full information on exposure, outcome and all covariates (n=28141) | Difference between study sample and study sample with full information  p-value^a^ |
| --- | --- | --- | --- | --- | --- |
|  | n (%) | n (%) |  | n (%) |  |
| Age |  |  | <0.001 |  | 0.808 |
| 2 years | 62922 (51.2) | 15472 (49.7) |  | 13952 (49.6) |  |
| 3 years | 59979 (48.8) | 15653 (50.3) |  | 14189 (50.4) |  |
| Total | 122901 | 31125 |  | 28141 |  |
| Child gender |  |  | 0.346 |  | 0.808 |
| Boy | 61170 (51.4) | 15916 (51.1) |  | 14405 (51.2) |  |
| Girl | 57806 (48.6) | 15209 (48.9) |  | 13736 (48.8) |  |
| Total | 118976 | 31125 |  | 28.141 |  |
| Gestation age |  |  | 0.052 |  | 0.608 |
| <37 weeks | 7159 (6.1) | 1743 (5.8) |  | 1554 (5.7) |  |
| ≤37 weeks | 109639 (93.9) | 28186 (94.1) |  | 25626 (94.3) |  |
| Total | 116798 | 29929 |  | 27180 |  |
| Family educational level |  |  |  |  |  |
| Short^c^ | 50026 (42.2) | 12851 (42.0) | 0.527 | 11536 (41.0) | 0.014 |
| Medium^d^ | 33762 (28.5) | 8584 (28.0) | 0.084 | 7872 (28.0) | 0.999 |
| Long^e^ | 34817 (29.3) | 9200 (30.3) | <0.001 | 8733 (31.0) | 0.066 |
| Total | 118605 | 30635 |  | 28141 |  |
| Parental employment status |  |  |  |  |  |
| Both parents in employment/work | 78891 (68.4) | 19000 (66.4) | <0.001 | 18803 (66.8) | 0.312 |
| One parent in employment/work | 28531 (24.7) | 7780 (27.2) | <0.001 | 7579 (26.9) | 0.421 |
| No parents in employment/work | 7945 (6.9) | 1833 (6.4) | 0.003 | 1759 (6.3) | 0.625 |
| Total | 115367 | 28613 |  | 28141 |  |
| Migration status |  |  |  |  |  |
| Both parents of Danish Origin | 86673 (73.3) | 21455 (74.7) | <0.001 | 21250 (75.5) | 0.027 |
| One parent Danish and one parent descendant of immigrants or immigrant | 11667 (9.9) | 3240 (11.3) | <0.001 | 3080 (10.9) | 0.129 |
| Both parents immigrants or descendants of immigrants | 19849 (16.8) | 4046 (14.1) | <0.001 | 3811 (13.5) | 0.038 |
| Total | 118189 | 28741 |  | 28141 |  |

^a^ Sample of children with available data on both exposure (mobile device screen time) and language development (language comprehension and expressive language skills), ^b^ chi2-test ; ^c^ short-cycle higher education, vocational school, high school or less, ^d^ medium-cycle higher education, ^e^ long-cycle higher education.
