# Supplementary material for: Mobile device screen time is associated with poorer language development among toddlers: results from a large-scale survey
Source: BMC Public Health. 2024 Apr 15;24:1050. doi: 10.1186/s12889-024-18447-4 (PMC11020890; doi:10.1186/s12889-024-18447-4)
Supplement: Supplementary file 2 — Supplementary Material 2 [file 12889_2024_18447_MOESM2_ESM.docx]

**Supplementary table S2**

*The FTF-Toddlers language comprehension subscale.*

| Item | Language comprehension subscale |
| --- | --- |
| 54 | Has difficulty understanding words |
| 55 | Has difficulty understanding simple instructions. |
| 56 | Has difficulty with opposite words like yes/no, happy/sad |
| 57 | Has difficulty with terms like big/small, in/on |
| 58 | Has difficulty remembering two instructions (e.g. put on your pajamas and go to the bed). |
| 59 | Has difficulty understanding a story he/she hears read aloud. |
| 60 | Has difficulty with the meaning of if – later (e.g. if you eat food now you will get ice cream later). |
