# Supplementary material for: Mobile device screen time is associated with poorer language development among toddlers: results from a large-scale survey
Source: BMC Public Health. 2024 Apr 15;24:1050. doi: 10.1186/s12889-024-18447-4 (PMC11020890; doi:10.1186/s12889-024-18447-4)
Supplement: Supplementary file 3 — Supplementary Material 3 [file 12889_2024_18447_MOESM3_ESM.docx]

**Supplementary table S3**

*The FTF-Toddlers spoken language subscale divided into the proposed subscales: expressive language skills and speech*

| Item | Spoken language subscale |
| --- | --- |
|  | *Expressive language skills* |
| 61 | Has difficulty saying single words and short sentences |
| 62 | Has difficulty speaking so that his/her parents understand him/her. |
| 63 | Has difficulty speaking so that strange people understand him/her. |
| 64 | Makes language sound mistakes (e.g. says t instead of f, like tota instead of sofa). |
| 65 | Has difficulty finding words or uses alternate words (e.g. says food instead of spoon). |
|  | *Speech related aspects* |
| 66 | Has a hoarse voice.* |
| 67 | Has a shrill voice.* |
| 68 | Stutters, or repeats words or parts of words over and over.* |
| 69 | Speaks so quickly that it is hard to comprehend what he/she is saying.* |
| 70 | Speaks very unclearly/mumbles.* |

* Items excluded from the scale used in the present study
