# Supplementary material for: Mobile device screen time is associated with poorer language development among toddlers: results from a large-scale survey
Source: BMC Public Health. 2024 Apr 15;24:1050. doi: 10.1186/s12889-024-18447-4 (PMC11020890; doi:10.1186/s12889-024-18447-4)
Supplement: Supplementary file 4 — Supplementary Material 4 [file 12889_2024_18447_MOESM4_ESM.docx]

**Supplementary table S4**

*Iterated principal factor analysis of the TFT-Toddlers spoken language scale with oblique (Promax) rotation.*

| Item | Factor 1  (Expressive language skills) | Factor 2  (speach related aspects) | Communality |
| --- | --- | --- | --- |
| Has difficulties saying single words and short sentences | 0.76 | 0.33 | 0.58 |
| Has difficulties speaking so that his/her parents understand him | 0.83 | 0.36 | 0.69 |
| Has difficulties speaking so that strange people understand him/her | 0.82 | 0.29 | 0.67 |
| Makes language sound mistakes (e.g. says t instead of f, like tota instead of sofa) | 0.64 | 0.25 | 0.41 |
| Has difficulties finding words or uses alternative words (e.g. says food instead of spoon) | 0.68 | 0.35 | 0.46 |
| Has a hoarse voice | 0.20 | 0.43 | 0.19 |
| Has a shrill voice | 0.20 | 0.48 | 0.23 |
| Stutters, or repeats word or parts of words over and over | 0.21 | 0.54 | 0.29 |
| Speaks so quickly that it is hard to comprehend what he/she is saying | 0.25 | 0.54 | 0.29 |
| Speaks very unclearly/mumbles | 0.51 | 0.49 | 0.34 |
| Eigenvalue | 3.36 | 0.79 |  |
| Variance explained | 80.8% | 19.2% |  |
