# Supplementary material for: Mobile device screen time is associated with poorer language development among toddlers: results from a large-scale survey
Source: BMC Public Health. 2024 Apr 15;24:1050. doi: 10.1186/s12889-024-18447-4 (PMC11020890; doi:10.1186/s12889-024-18447-4)
Supplement: Supplementary file 5 — Supplementary Material 5 [file 12889_2024_18447_MOESM5_ESM.docx]

**Supplementary table S5**

*FTF-Toddlers cut-off scores (90-percentile) for language comprehension and expressive language skills difficulties used in the present study*

|  |  | Scoring Range | 90% Cut-of score | Risk of difficulties (n) |
| --- | --- | --- | --- | --- |
| *Language comprehension* |  |  |  |  |
| 2 years | Boys | 0-14 | ≥7 | 725 |
| 2 years | Girls | 0-14 | ≥5 | 937 |
| 3 years | Boys | 0-14 | ≥4 | 936 |
| 3 years | Girls | 0-14 | ≥4 | 720 |
| *Expressive language skills* |  |  |  |  |
| 2 years | Boys | 0-10 | ≥8 | 804 |
| 2 years | Girls | 0-10 | ≥6 | 870 |
| 3 years | Boys | 0-10 | ≥5 | 806 |
| 3 years | Girls | 0-10 | ≥4 | 737 |
